# Effect of Fast Freeze-Thaw Cycles on Mechanical Properties of Ordinary-Air-Entrained Concrete

**DOI:** 10.1155/2014/923032

**Published:** 2014-05-07

**Authors:** Huai-shuai Shang, Wei-qun Cao, Bin Wang

**Affiliations:** ^1^State Key Laboratory of Simulation and Regulation of Water Cycle in River Basin, China Institute of Water Resources and Hydropower Research, Beijing 100038, China; ^2^Collaborative Innovation Center of Engineering Construction and Safety in Shandong Blue Economic Zone, Qingdao Technological University, Qingdao, Shandong 266033, China

## Abstract

Freezing-thawing resistance is a very significant characteristic for concrete in severe environment (such as cold region with the lowest temperature below 0°C). In this study, ordinary-air-entrained (O-A-E) concrete was produced in a laboratory environment; the compressive strength, cubic compressive strength of C50, C40, C30, C25, and C20 ordinary-air-entrained concrete, tensile strength, and cleavage strength of C30 ordinary-air-entrained concrete were measured after fast freeze-thaw cycles. The effects of fast freeze-thaw cycles on the mechanical properties (compressive strength and cleavage strength) of ordinary-air-entrained concrete materials are investigated on the basis of the experimental results. And the concise mathematical formula between mechanical behavior and number of fast freeze-thaw cycles was established. The experiment results can be used as a reference in design, maintenance, and life prediction of ordinary-air-entrained concrete structure (such as dam, offshore platform, etc.) in cold regions.

## 1. Introduction

Durability of concrete [1–4] is the ability to retain its original form and quality without significant deterioration for a long time. Factors [5–8] causing the damage of concrete material in structure can be divided into two categories: physical effects (such as freeze-thaw damage and abrasion) and chemical effects (such as sulfate attack and corrosion of reinforcing steel), in the whole design life. As a widely used construction material, the durability characteristics of concrete are all significant to its sustained use. Due to the need of practical application, many reinforced concrete structure were (will be) built in cold regions that inevitably subjected to freezing and thawing action [9–12]. One main reason of durability problem in reinforced concrete structures in cold environment is the damage caused by action of freezing and thawing.

The effects of action of freeze/thaw cycles on air-entrained concrete and plain concrete are well documented and many researchers have documented the improvement on the freeze/thaw resistance of air-entrained concrete over plain concrete [13–19]. Reference [13] introduced the experiment study about freezing and thawing resistance of air-entrained concrete in which the coarse aggregate was produced from air-entrained concrete and non-air-entrained concrete, respectively. Reference [17] investigated behavior (strength and durability) of air-entrained CSF (condensed silica fume) concrete after the action of freezing and thawing cycles. The author of this paper studied the strength of air-entrained concrete under multiaxial compression, multiaxial tension-compression, uniaxial compression, uniaxial tension prior to freeze-thaw cycles and weight loss, and dynamic modulus of elasticity and strength under tensile load, cleavage load, and compressive load after different cycles of fast freeze-thaw cycles [18, 19].

Many current research works were found to deal with the durability of concrete, especially the freeze-thaw resistance in cold environment [9–12, 18, 19]. However, this question still needed further investigations. As one of the main measures to improve the frost resistance of concrete, ordinary-air-entrained concrete has been applied to many kinds in civil engineering in cold regions and has got very obvious economic benefits and social effects. Present works often focused on the impact of freeze-thaw action on relative dynamic modulus of elasticity and weight loss of air-entrained concrete; very little work reported about the mechanical behavior of O-A-E concrete after fast freeze-thaw cycles were formed. So the systemic experimental study of O-A-E concrete after fast freeze-thaw cycles was presented in this paper. The mechanical properties (compressive strength, cubic compressive strength, and cleavage strength) of C50, C40, C30, C25, and C20 O-A-E concrete after fast freeze-thaw cycles were studied according to the test method of long-term and durability on ordinary concrete GB/T 50082-2009 [20].

## 2. Experimental Procedures

### 2.1. Materials and Mix Proportions

In this study, local materials (1) Portland cement 32.5^#^ and 42.5^#^ (GB175-2007) [21] (the standard compressive strength of 32.5^#^ and 42.5^#^, Portland cement was 32.5 and 42.5 MPa at 28 days), (2) natural river sand (fineness modulus was 2.6), and (3) coarse aggregate (crushed stone with diameter 5 mm~10 mm) were used.  Table 1 lists the mix proportion of C50, C40, C30, C25, and C20 ordinary-air-entrained concrete by weight. The mixing was accomplished by putting all the coarse and fine aggregates into the mixer. These ingredients were mixed for about 1 min, and then the water was added in 1 minute. The mixing continued for about 2 min after all water was added.

### 2.2. Test Specimens and Testing Programs

The concrete samples used for cubic compressive strength tests were cube with dimensions: 100 mm × 100 mm × 100 mm. The concrete samples used for compressive strength tests were prismoid with dimensions: 100 mm × 100 mm × 200 mm (got through severing the prismoid specimen from middle). Steel molds were used to cast the concrete samples. All concrete samples were demoulded at 24 h after casting. Thereafter, all the samples were cured in the condition with 20 ± 3°C and at 95% RH for 23 days. Then the samples were stored in water (the time in water was 4 days) until the test of fast freezing and thawing cycles.

In this study, the freeze-thaw test machine [22] meeting the national stand GB/T 50082-2009 requirement was used. The freeze-thaw test machine consists of 28 rubber barrels in which specimens were completely immersed by water. The “freeze” process in fast freeze-thaw cycle is reducing the temperature of the O-A-C concrete samples from 6°C to −15°C; the “thaw” process is raising the temperature from −15°C to 6°C, while the temperature of the coolant reduces from 8 ± 2°C to −17 ± 2°C in the “freeze” process and is raised from −15°C to 6°C in the “thaw” process all in about 2.5~3 hours.

**Figure 1:**
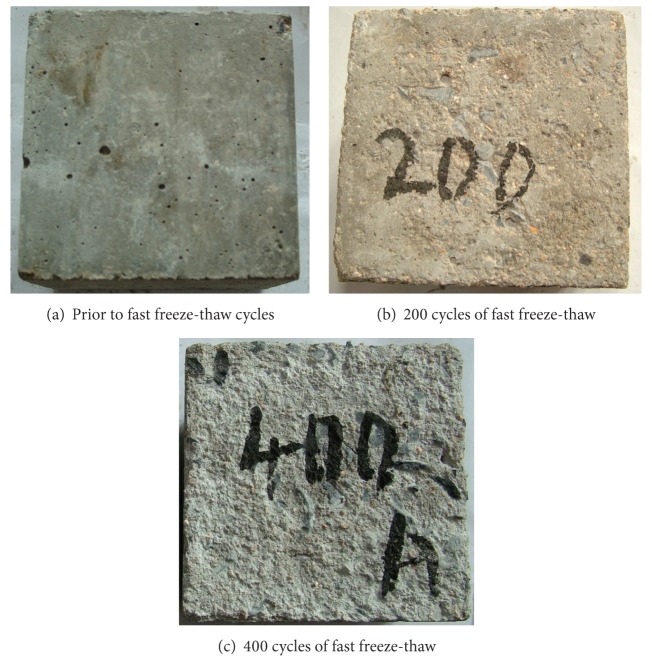
Surface of O-A-C concrete after fast freeze-thaw cycles.

The deterioration of surface of O-A-C concrete samples undergoing fast freeze-thaw cycles is shown in Figure 1. Just as shown in Figure 1, the specimen surface was gradually damaged and cement slurry gradually peeled from the surface as the number of fast freeze-thaw cycles increased. The uneven surface was caused by the action offast freeze-thaw cycles, and surface damage would get worse withfast freeze-thaw cycles increased. In addition to the fact that the spalling of part was serious, the spalling of remaining surface was rare after 200 fast freeze-thaw cycles, while after 400 cycles of fast freeze-thaw, most of the mortar in surface has stripped andpart of the aggregate was exposed. Compared with the damage of plain concrete, the damage of O-A-E concrete is relatively light under the action of the same number of fast freeze-thaw cycles.

## 3. Results and Discussions

### 3.1. Failure Modes

**Figure 2:**
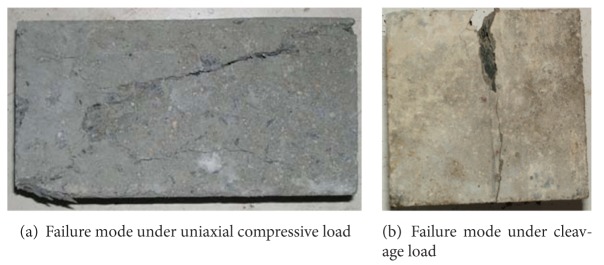
Failure modes of O-A-C concrete samples.

Figure 2(a) shows the failure modes of O-A-C concrete samples under uniaxial compressive loading.  Figure 2(b) shows the failure modes of O-A-C concrete samples under cleavage loading [23]. The above two kinds of failure modes were mainly due to the tensile strain along the free surface(s). It was obvious that the action of the fast freeze-thaw cycles did not modify the tensile splitting mode. There was no difference in the failure modes for C50, C40, C30, C25, and C20 ordinary-air-entrained concrete after fast freeze-thaw cycles.

The tensile strain in the direction perpendicular to free surface will be caused due to compression load or cleavage load. And the crack(s) will be generated when the tensile strain exceeds the ultimate tensile strain. It was noticed that direction of the crack(s) is random due to the impact of the uneven distribution of coarse aggregates. The failure process of the O-A-C concrete samples under the action of cleavage and tension load was instantaneous and was always caused by a single crack at the center of the sample.

### 3.2. The Cubic Compressive Strength and Compressive Strength

For the concrete structures in cold region, the deterioration of concrete material was mainly caused by internal crack growth due to fast freeze-thaw cycles. The loss of strength will be caused during fast freeze-thaw cycles. So the mechanical properties of ordinary-air-entrained concrete after fast freeze-thaw cycles were measured.

The compressive stress was got through dividing the applied load by loading-carrying area (0.01 m^2^); at least three sample tests were carried out for each test group. Tables 2 and 3 list the experiment results of mechanical behavior (compressive strength and cubic compressive strength) of C50, C40, C30, C25, and C20 ordinary-air-entrained concrete after fast freeze-thaw cycles, respectively.

**Figure 3:**
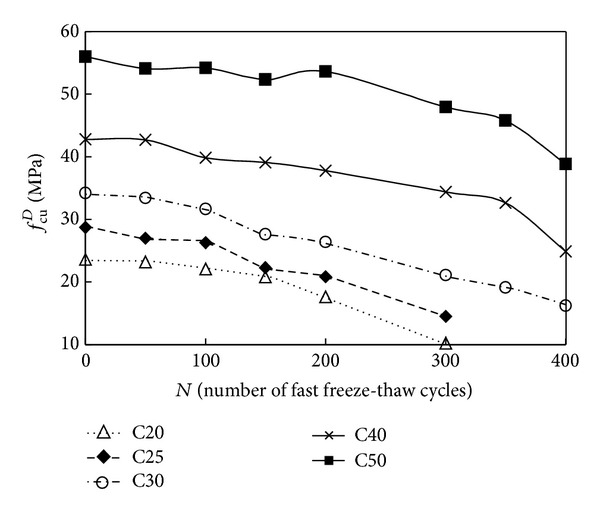
Number of fast freeze-thaw cycles versus the cubic compressive strength of O-A-E concrete.

**Figure 4:**
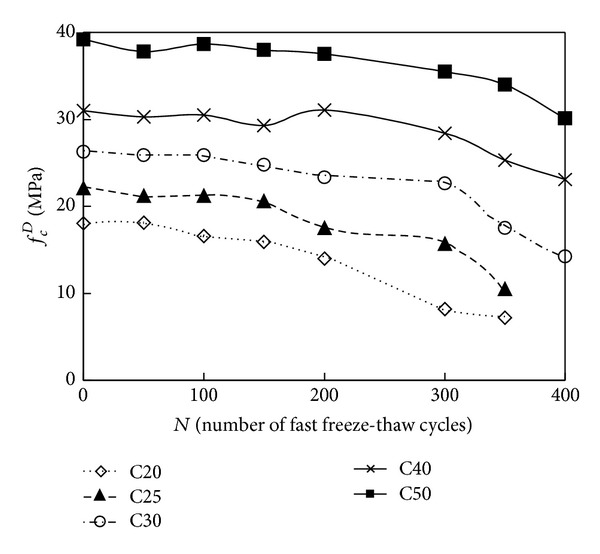
Number of fast freeze-thaw cycles versus the compressive strength of O-A-E concrete.

The relationship of the cubic compressive strength versus number of fast freeze-thaw cycles and compressive strength versus number of fast freeze-thaw cycles is shown in Figures 3 and 4. As seen from Tables 2 and 3 and Figures 3 and 4, the value of mechanical property (cubic compressive strength and compressive strength) decreased as the number of fast freeze-thaw cycles increased. After the action of the same cycles of fast freeze-thaw, the decreased percentage of the value of cubic compressive strength and compressive strength is larger for C20 O-A-E concrete samples than for C50, C40, C30, and C25 O-A-E concrete samples. After the action of 300 cycles of fast freeze-thaw, the cubic compressive strength of C50, C40, C30, C25, and C20 O-A-E concrete decreased to 85.6, 80.3, 61.7, 50.5, and 43.3 percent of the initial value of cubic compressive strength, while the compressive strength of C50, C40, C30, C25, and C20 O-A-E concrete decreased to 90.6, 91.6, 86.0, 70.0, and 45.4 percent of the initial value.

Reference [24, 25] investigated the relationship between number of fast freeze-thaw cycles and mechanical property of plain concrete, respectively. According to the experiment results in [24], the compressive strength value after 100 cycles of fast freeze-thaw decreased to 44.4 percent of the initial value prior to fast freeze-thaw cycles, while the result of the compressive strength value after 125 cycles of fast freeze-thaw decreased to 61.0 percent of the initial value prior to fast freeze-thaw cycles was got in [25].

**Figure 5:**
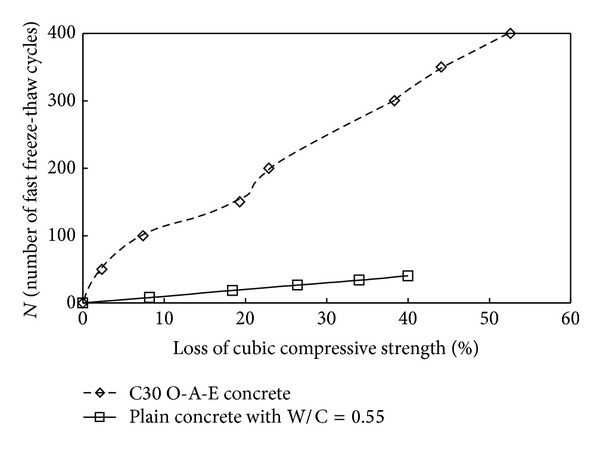
Influence of fast freeze-thaw cycles on the strength of plain concrete and O-A-E concrete.

Figure 5 shows the effect of fast freeze-thaw cycles on the strength of plain concrete and O-A-E concrete cubic specimens under compression after fast freeze-thaw cycles. Just as shown in Figure 4, the compression strength loss of plain concrete specimen is larger than that of O-A-E concrete after the action of the same cycles of freeze-thaw. It means that, compared with plain concrete, O-A-E concrete can withstand more cycles of fast freeze-thaw. For plain concrete specimen, the cubic compressive strength after 75 cycles of fast freeze-thaw decreased to 60.0% of its initial value, while, for O-A-E concrete, after the action of 300 cycles of fast freeze-thaw, the cubic compressive strength decreased to 61.7% of its initial value.

### 3.3. The Cleavage Strength

The cleavage strength can be drawn through [23] as follows:

(1) $$\begin{array}{c} f_{ts}=\frac{2F}{\pi A}=0.637\frac{F}{A}, \end{array}$$

where *f*_*ts*_ denotes the cleavage strength, *F* is the failure load, and *A* is the area under cleavage load.

Table 4 gives the cleavage strength of C30 O-A-E concrete after fast freeze-thaw cycles.

As seen from Table 4, the cleavage strength decreased to 90.4 and 55.2 percent after 100 and 400 cycles of freeze-thaw, respectively. For plain concrete [12], only after 50 cycles of fast freeze-thaw, the cleavage strength has been decreased to 55.4 percent. The behavior of O-A-E concrete specimen under uniaxial tensile was studied and discussed by the author in [18]. According to the experimental results, for O-A-E concrete, the direct tensile strength was 10.1 percent of the compressive strength prior to fast freeze-thaw cycles, while, after 400 cycles of fast freeze-thaw, the direct tensile strength was 6.9 of the compressive strength. Reference [26] studied the effect of fast freeze-thaw cycles on the tensile strength of air-entrained high-strength concrete. It found that the value of direct tensile strength was 4.2 percent of the compressive strength prior to fast freeze-thaw cycles and 3.4 percent of the compressive strength after 700 cycles of freeze-thaw.

The relationship between the cubic compressive strength (compressive strength) and numbers of fast freeze-thaw cycles was analyzed through the least square regression method; the mathematic expression of exponential function is given out as

(2) fcuDfcu(fcDfc)=α1(β1)+α2(β2)×e(−0.01×N) +α3(β3)×0.01×N×e(−0.01×N),

where *f*_cu_^*D*^(*f*_*c*_^*D*^) is the value of cubic compressive strength (compressive strength) of O-A-C concrete after fast freeze-thaw cycles; *N* is the number of fast freeze-thaw cycles; *α*_1_,  *α*_2_, and *α*_3_  are the regress parameters for the relationship between cubic compressive strength and number of fast freeze-thaw cycles; *β*_1_, *β*_2_, and *β*_3_ are the regress parameters for the relationship between compressive strength and number of fast freeze-thaw cycles.

The value ofregress parameters *α*_1_, *α*_2_, *α*_3_, *β*_1_, *β*_2_, and *β*_3_ was given in Table 5 through calculating analysis.

**Figure 6:**
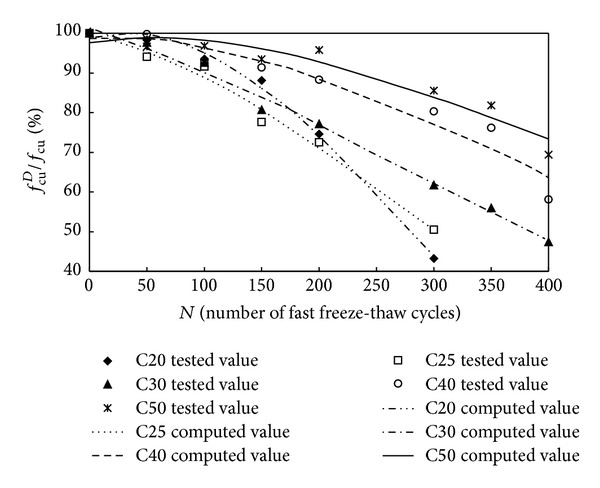
Relationship of the cubic compressive strength versus freeze-thaw cycles.

**Figure 7:**
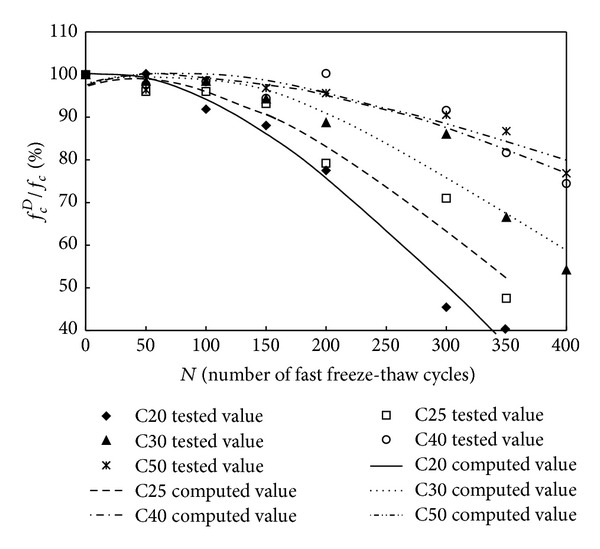
Relationship of the compressive strength versus freeze-thaw cycles.

For the cubic compressive strength and compressive strength of O-A-E concrete after the action of fast freeze-thaw cycles, a comparison of the experimental results with calculated results obtained from (2) is illustrated in Figures 6 and 7. As shown in Figures 6 and 7, the calculated results of cubic compressive strength and compressive strength according to (2) are in good agreement with the experimental data.

### 3.4. Strength Loss

**Figure 8:**
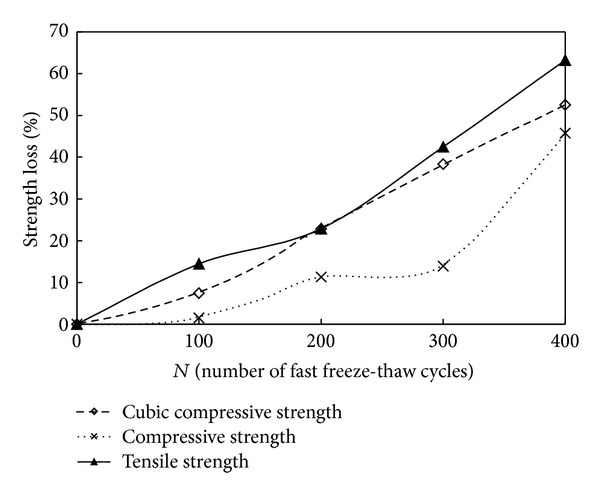
The strength loss of air-entrained concrete versus number of freeze-thaw cycles.

Figure 8 gives the strength (the strength under uniaxial compression with cube specimens and prismoid specimens) loss of C30 O-A-E concrete when subjected to action of fast freeze-thaw cycles. The loss of compressive strength, cubic compressive strength, and tensile strength was 1.5, 2.3, and 14.5 percent after 100 cycles of fast freeze-thaw for C30 O-A-E concrete and 14.0, 38.3, and 42.4 percent after 300 cycles of fast freeze-thaw, respectively. Compared to the loss of tensile strength and cubic compressive strength, the loss of compressive strength was more moderate, especially in the first 300 cycles of fast freeze-thaw.

It can be concluded that there was no great difference in the strength loss of O-A-E concrete under uniaxial tension and compression due to fast freeze-thaw cycles. But the reduction of compressive strength can be associated with the initiation and propagation of crack parallel to the direction of tensile load due to the action of fast freeze-thaw cycles. However, the reduction of tensile strength should be associated with the initiation and propagation of crack perpendicular to the direction of tensile load.

For O-A-E concrete under the action of tensile load, the initiation and growth of inherent microcracks leaded to the decrease of carrying area, and then the stress concentration at critical crack tips will be caused and at last resulted in the cracks to propagate further. So damage even failure of the concrete occurs after the initiation and growth of crack caused by fast freeze-thaw cycles.

### 3.5. Discussion

The durability of O-A-E concrete was improved through reducing stresses caused by freezing water in pores. The volume expansion when water converts from liquid to solid under the action of “freezing.” The relief for this pressure can be obtained when entrained air was introduced into concrete which can provide space for free water to flow into. But if there were no air voids, the stress will be caused by pressure on the concrete and the stress can create cracks which cumulatively begin to cause disruption of the concrete.

Plain concrete also contains entrapped air that exists with larger bubbles. Compared with bubble state in O-A-E concrete, the larger bubbles in plain concrete are typically less evenly distributed. And these larger bubbles cannot effectively reduce stress caused by the action of “freezing.” Through comparing the test results in this paper with the conclusion got by other authors [22, 24], the conclusion that the strength loss rate of ordinary-air-entrained concrete becomes notably lower than that of plain concrete with the increasing of fast freeze-thaw cycles is got. It means that the durability deterioration of ordinary-air-entrained concrete is slower than that of plain concrete. It is because the mixed air-entraining agent in concrete can make them up effectively and thus improve the durability.

## 4. Conclusion

Based on the experimental work in this paper and the discussion about the test results, the following conclusions can be drawn.

1. The mechanical property (cleavage strength, compressive strength, and tensile strength) of C30 O-A-E concrete decreased as the fast freeze-thaw cycles were repeated; the strength loss of C30 O-A-E concrete under compression and tension under the action of fast freeze-thaw cycles is evident. After the same cycles of fast freeze-thaw, the decreased percentage of cubic compressive strength and compressive strength is larger for C20 O-A-E concrete specimens than for C25, C30, C40, and C50 O-A-E concrete specimens.
2. The main reason that induced cracks in concrete is the volume expansion caused by water frozen into ice. The second reason that induced cracks is the thermal stress developed under the action of repeated freeze-thaw.
3. The durability of concrete can be improved greatly by adding air-entraining agent into concrete.
4. The freeze-thaw durability of concrete should be taken into consideration in structure design and maintenance.

## Figures and Tables

**Table 1:** The mix proportion of air-entrained concrete in per cubic meter.

	W/C	Cement	Cement (kg/m^3^)	Water (kg/m^3^)	Sand (kg/m^3^)	Coarse aggregate (kg/m^3^)	Air-entraining dgent (kg/m^3^)	Air content (%)
C50	0.32	42.5	526.00	168.30	520.00	1154.80	1.30	5.5~6.5
C40	0.36	42.5	467.60	166.00	568.20	1148.00	1.17	5.5~6.5
C30	0.40	42.5	412.67	164.30	586.83	1186.00	1.03	5.5~6.5
C25	0.40	32.5	356.00	141.00	615.20	1188.00	0.89	5.5~6.5
C20	0.40	32.5	339.00	133.80	642.00	1185.20	0.85	5.5~6.5

**Table 2:** Cubic compressive strength of O-A-E concrete after fast freeze-thaw cycles.

	Number of fast freeze-thaw cycles							
	0	50	100	150	200	300	350	400
C50	55.98	54.12	54.20	52.33	53.62	47.92	45.80	38.85
C40	42.80	42.70	39.86	39.11	37.79	34.38	32.61	24.88
C30	34.20	33.40	31.67	27.60	26.38	21.10	19.13	16.22
C25	28.70	27.00	26.30	22.28	20.82	14.50	/	/
C20	23.60	23.20	22.07	20.80	17.60	10.21	/	/

**Table 3:** Compressive strength of O-A-E concrete after fast freeze-thaw cycles.

	Number of fast freeze-thaw cycles							
	0	50	100	150	200	300	350	400
C50	39.19	37.80	38.66	37.96	37.50	35.50	33.99	30.12
C40	31.02	30.30	30.52	29.30	31.10	28.42	25.33	23.10
C30	26.30	25.90	25.90	24.80	23.33	22.63	17.50	14.25
C25	22.10	21.22	21.22	20.60	17.50	15.69	10.50	/
C20	18.06	18.10	16.60	15.90	14.00	8.20	7.20	/

**Table 4:** Cleavage strength of C30 O-A-E concrete after fast freeze-thaw cycles (MPa).

Number of fast freeze-thaw cycles	0	100	200	300	400
Cleavage strength	2.81	2.54	2.35	2.15	1.55

**Table 5:** The value of *α*_1_, *α*_2_, *α*_3_, *β*_1_, *β*_2_, and *β*_3_ after different freeze-thaw cycles.

	Cubic compressive strength			Compressive strength		
	*α*_1_	*α*_2_	*α*_3_	*β*_1_	*β*_2_	*β*_3_
C50	−0.21	1.19	1.38	0.04	0.94	1.13
C40	−0.42	1.41	1.48	−0.21	1.19	1.48
C30	−0.53	1.54	1.20	−1.01	1.99	2.35
C25	−1.33	2.32	2.08	−1.52	2.50	2.74
C20	−2.84	3.83	4.15	−2.09	3.09	3.20
